# Heat-Related Illness in Emergency and Critical Care: Recommendations for Recognition and Management with Medico-Legal Considerations

**DOI:** 10.3390/biomedicines10102542

**Published:** 2022-10-12

**Authors:** Gabriele Savioli, Christian Zanza, Yaroslava Longhitano, Alba Nardone, Angelica Varesi, Iride Francesca Ceresa, Alice Chiara Manetti, Gianpietro Volonnino, Aniello Maiese, Raffaele La Russa

**Affiliations:** 1 Emergency Department, IRCCS Policlinico San Matteo, 27100 Pavia, Italy; 2 Doctoral Program Experimental Medicine, Department of Clinical-Surgical, Diagnostic and Pediatric Sciences, University of Pavia, 27100 Pavia, Italy; 3 Foundation “Ospedale Alba-Bra”, Department of Emergency Medicine, Anesthesia and Critical Care Medicine, Michele and Pietro Ferrero Hospital, 12060 Verduno, Italy; 4 Department of Internal Medicine, Università degli Studi of Pavia, 27100 Pavia, Italy; 5 Department of Emergency Medicine, Ospedale Civile of Vigevano, 27029 Vigevano, Italy; 6 Department of Surgical, Medical and Molecular Pathology and Critical Care Medicine, Institute of Legal Medicine, University of Pisa, 56126 Pisa, Italy; 7 Department of Anatomy, Histology, Forensic Medicine and Orthopedics, Sapienza University, 00185 Rome, Italy; 8 Department of Clinical and Experimental Medicine, University of Foggia, 71122 Foggia, Italy

**Keywords:** environmental heat emergency, heat emergency, heat stroke, hyperthermia

## Abstract

Hyperthermia is an internal body temperature increase above 40.5 °C; normally internal body temperature is kept constant through natural homeostatic mechanisms. Heat-related illnesses occur due to exposure to high environmental temperatures in conditions in which an organism is unable to maintain adequate homeostasis. This can happen, for example, when the organism is unable to dissipate heat adequately. Heat dissipation occurs through evaporation, conduction, convection, and radiation. Heat disease exhibits a continuum of signs and symptoms ranging from minor to major clinical pictures. Minor clinical pictures include cramps, syncope, edema, tetany, and exhaustion. Major clinical pictures include heatstroke and life-threatening heat stroke and typically are expressed in the presence of an extremely high body temperature. There are also some categories of people at greater risk of developing these diseases, due to exposure in particular geographic areas (e.g., hot humid environments), to unchangeable predisposing conditions (e.g., advanced age, young age (i.e., children), diabetes, skin disease with reduced sweating), to modifiable risk factors (e.g., alcoholism, excessive exercise, infections), to partially modifiable risk factors (obesity), to certain types of professional activity (e.g., athletes, military personnel, and outdoor laborers) or to the effects of drug treatment (e.g., beta-blockers, anticholinergics, diuretics). Heat-related illness is largely preventable.

## 1. Epidemiology

Heat illness is a commonly occurring disorder around the world, according to clinical research institutes, it is the leading cause of climate-related deaths, and its incidence is increasing. In the United States, according to some authors, it represents the third leading cause of death for high school athletes [1]. These data are corroborated by the Center for Disease Control (CDC) [2].

In the United States, between 2000 and 2010, there were over 30,000 hospitalizations for heat diseases, with a predominance of cases amongst the elderly, athletes, and outdoor workers. ED-visits in the same years were 5 per 10,000 mostly comprising of heat exhaustion (75%), with major clinical pictures representing about 5% of cases, and with mortality of less than 0.1% [3,4]. The regions most affected were the mid-west and the south, which have constant exposure to higher temperatures throughout the year. The number of hospitalizations depended on the maximum monthly temperature [5].

The incidence of heat-related illness in U.S. high school athletes is approximately 9000 cases annually [6].

These diseases also have a high prevalence in the U.S. armed forces, with an incidence of 1000 person-years, incidence has gradually increased since 2014 [7].

There are millions of annual presentations of minor clinical pictures. It can, therefore, be said that heat exhaustion frequently represents the first stage in a continuum of pathologic states that typically affect more fragile groups of people, or those more exposed to exercise or prolonged exposure in a hot humid environment, for example, for work reasons [8,9,10,11,12,13].

## 2. Risk Factors

Heat accumulation is determined by a combination of mechanisms, such as exposure to a humid hot environment, metabolic production of heat, and dysfunction of heat dissipation mechanisms. Under these conditions, temperature homeostasis is compromised resulting in clinical symptoms.

Although the major risk factors include strenuous exercise in a hot humid climate in the absence of acclimatization, all risk factors must be carefully considered [10,14,15].

There are some categories of people at greater risk of developing these diseases, due to exposure in certain geographical areas (e.g., hot humid environments), unmodifiable predisposing conditions (e.g., old age, childhood, diabetes, skin diseases with reduced sweating), modifiable risk factors (e.g., alcoholism, excessive exercise, infections), partially modifiable risk factors (e.g., obesity), membership of certain professional categories (e.g., athletes, military personnel, and outdoor laborers), or drug-treatment (e.g., beta-blockers, anticholinergics, diuretics). However, heat-related illness is largely preventable: even the most vulnerable subjects with non-modifiable risk factors can protect themselves by avoiding hazardous exposure, and all modifiable or partially modifiable risk factors can be modulated and monitored to avoid the onset of disease from heat.

### 2.1. Non-Modifiable Risk Factors

Among non-modifiable risk factors (Table 1), the most relevant is age. Elderly patients are more at risk due to reduced cardiovascular physiological reserves, an impaired sense of thirst, and compromised regulatory systems due to senile degeneration. In addition, they have reduced tolerance to orthostasis and often take various drugs, including diuretics, beta-blockers, and antidepressants. Children are more at risk because of an increased body surface index, and they are often dependent on others to satisfy their thirst, as for geriatric people.

Non-modifiable risk factors are chronic or acute pathologies including.

Dermis diseases (such as scleroderma and burns) that compromise heat dissipation. In such patients, the ability to dissipate heat and maintain adequate hydration is impaired, so those affected are more vulnerable to heat disease even during brief exposure to elevated ambient temperature or when performing exercise in moderate heat stress conditions [16,17]. Autonomic disorders that cause widespread anhidrosis. Sweating is one of the main mechanisms through which the organism can tolerate high temperatures if lost fluids are adequately replaced. When sweating is impaired, there is an increased predisposition to heat sickness. Pathologies include acquired idiopathic generalized anhidrosis, Ross syndrome, chronic idiopathic anhidrosis, and Sjögren syndrome. Patients with these conditions are more susceptible to heat stress [18].

Diabetes mellitus involves multiple mechanisms which can impair cutaneous circulation and vasa nervorum, and, with the development of neuropathy, may increase the potential for developing hyperthermia [19,20].

Severe traumatic pathology like spinal cord transection may also impair thermoregulation [21,22].

Malignant hyperthermia is a hereditary disease that impairs the regulation of calcium in skeletal muscle through uncontrolled skeletal muscle hypermetabolism that causes hyperthermia, muscle stiffness, tachycardia, acidosis, and hyperkalemia following certain environmental triggers [23].

Furthermore, prolonged epileptic seizures in a warm humid environment or under critical external conditions, as well as decompensated endocrinological pathologies (e.g., hyperthyroidism, in which there is an increased production of body heat) may contribute to hyperthermia [24].

Gender is another non-modifiable risk factor. Hyperthermia seems to occur more frequently in males, but this could be because a greater number of males engage in activities that predispose them to developing hyperthermia, such as manual and/or outdoor work.

### 2.2. Modifiable Risk Factors

Among modifiable risk factors (Table 1), alcoholism and intake of some drugs, such as carbonic anhydrase inhibitors (e.g., topiramate, acetazolamide) [25], M3 anticholinergic agents (e.g., bladder antispasmodics, tricyclic antidepressants, and neuroleptics) have a causal role [26,27,28]. For patients who arrive in an emergency department with suspicion of serotonin syndrome vital parameters must be monitored; hyperthermia is one of the earliest and most important signs of this syndrome. It occurs in patients taking serotonin-selective reuptake inhibitors multiply or in combination with other drugs, such as meperidine, fentanyl, tramadol, that enhance serotonin availability in the brain [29].

The recreational abuse of alcohol, psychomotor stimulants (e.g., cocaine, amphetamines, and derivatives including ecstasy), and/or heroin can cause hyperthermia.

There also is an idiosyncratic condition—neuroleptic malignant syndrome that is a condition very serious and can lead to death, albeit in less than 0.5% of cases. It is caused by taking dopamine 2 receptor antagonists, in particular haloperidol, aripiprazole, or lupentixol [30].

Some occupational categories, partly already mentioned above, are at higher risk of hyperthermia. These include military personnel, athletes, builder, field, mining or wellness and health workers, who combine physical exertion in a hot environment with limited airflow.

Obesity causes the impairment of heat dissipation capacity, so represents another modifiable, or partially modifiable, risk factor.

**Table 1:** Risk factors for heat related disease.

Non-Modifiable Risk Factors	Modifiable Risk Factors
Age (geriatric patients or children)	Dehydration
Autonomic disorders that cause widespread anhidrosis (Ross syndrome, chronic idiopathic anhidrosis, Sjögren syndrome)	Prolonged exposure in a warm humid environment
Trauma with spine injuries	Occupational categories (military personnel, athletes, construction, field, mining, or well workers, etc.)
Endocrinological disorders (diabetes, hyperthyroidism)	Addictive behaviors (alcoholism, cocaine, amphetamine, heroin use, etc.)
Neurological disorders (epilepsy)	Drugs (anticholinergics, beta-blockers, diuretics, neuroleptics, anesthetics, topiramate)
Skin diseases (scleroderma, burns)	Infections
Hereditary disease (malignant hyperthermia)	Obesity

## 3. Pathophysiology

Body temperature is kept constant, over certain physiological intervals, despite external and environmental factors affecting body heat, through a regulated balance between the production of heat and its dispersion.

### 3.1. Production of Heat

The production of heat depends on continuous metabolic activities in which, in basal conditions, thyroid thermogenesis and the action of ATPase are the main determinants. Muscle contraction and digestive processes are other sources of heat.

### 3.2. Heat Dispersion

Heat dispersion occurs through four mechanisms: conduction, convection, evaporation, and radiation [31].

1. Conduction is the transfer of heat to a cooler object through direct contact.
2. Convection is the transfer of heat at the body surface by air circulation.
3. Evaporation cools the skin surfaces when sweat changes from a liquid to a vapor
4. Radiation occurs through the transmission of electromagnetic waves.

Without mechanisms of heat dispersion, body temperatures could increase by as much as 1° C per hour.

However, each of these mechanisms depends on external conditions, such as the ambient temperature, presence of wind, direct sunlight, body immersion and environmental humidity.

The most immediate mechanism for regulating heat dissipation is increase in cutaneous and subcutaneous blood flow which enables dispersion of heat transported to the surface.

At high ambient temperatures and with increased metabolic demands, the evaporation of sweat is the primary mechanism for heat dissipation. It enables evaporation of up to one liter of sweat per hour but is highly limited in the event of high environmental humidity. Sweating enables toleration of high temperatures only where hydro-electrolytic homeostasis is maintained and sodium chloride and water are replaced.

### 3.3. Regulatory System

The regulatory system is complex and is mainly based on negative feedback. It consists of receptors in the skin and visceral organs that are sensitive to core temperatures, and a central integrative area, the hypothalamus, which is responsible for thermoregulation through a series of effectors, sweat, metabolic or vasomotor effectors, that are activated to favor the conservation or dispersal of heat. Information on temperature is obtained from thermal receptors located in the skin, aorta, arteries, and brain. The thermoregulation mechanisms, when effective, allow the internal temperature to vary around very small oscillations. When the temperature is normal, more than 50% of the heat produced is dispersed by radiation and a further third by sweating or breathing. When the ambient or body temperature exceeds 37 °C, heat dispersion occurs mainly through evaporation or sweating. High intracellular temperature damages cell membranes, enzymatic systems, mitochondria, and protein-coding systems. The damage is aggravated by the relative hypoxia that develops due to the increase in metabolic demands.

This complex adaptive response results in the following physiological reaction:

#### 3.3.1. Cutaneous System

Cutaneous vasodilation is regulated by the hypothalamus and increases the thermal conductivity of the skin, enhancing heat dispersion. Sweating is, alternatively, stimulated by cholinergic sympathetic innervation.

#### 3.3.2. Cardiovascular System

Heat stimulus causes an increase in cardiac output to increase blood and subcutaneous flow and to address perfusion stress [32,33,34].

With increased temperature, the heart rate also increases both for the sinoatrial node and cardiac conduction pathway, and via predominantly parasympathetic neural influences [35,36]. The stroke volume is less sensitive to heat stress despite increased cardiac contractility indices, but it may decrease in sudden or prolonged orthostatism [32,37,38,39,40].

## 4. Left Ventricular Preload and Afterload

High temperature increases cause a reduction in the pulmonary capillary wedge pressure and left-ventricular end-diastolic volume [35]. During mild heat stress, the mean arterial pressure is maintained, but it can decrease during more severe heating [35,41]. Heat stress decreases systemic vascular resistance, primarily due to large decreases in cutaneous vascular resistance.

## 5. Blood Volume and Coagulation

Heat stress results in a redistribution of body blood flow to the skin and subcutis with a consequential reduction in blood flow in other body areas and organs (e.g., brain, heart, liver, spleen, and kidneys) [37]. This allows for better heat dissipation. Some studies have shown that the increase in heat causes a decrease in activated partial thromboplastin time, thromboelastographic reaction time, and platelet count, as well as an increase in fibrinolytic activity [42,43]. There is therefore an early activation of the coagulation system when the body is exposed to heat stress.

## 6. Renal System

Heat stress induces a decrease in renal blood flow and increase in renal vascular resistance for intra-kidney blood flow redistribution. There is activation of vasoactive substances, such as norepinephrine and angiotensin II. Both systems are also activated by prolonged or sudden standing and physical exercise; therefore, these conditions, associated with heat stress, can contribute to further decrease in renal blood flow [44].

## 7. Brain and Cerebral Circulation

Cerebral blood flow decreases during heat stress [45]. Cerebral vascular resistance measured at the middle cerebral artery increases with heat stress [46]. These changes appear to be related to multiple factors, including temperature, sympathetic activity, and the partial pressure of arterial carbon dioxide [47].

## 8. Cytokines

Heat stress determines the activation of systemic inflammation, particularly through the activity of cytokines (e.g., IL-1β, interferon-γ, IL-6, and IL-10) contributing to multiorgan failure [48,49].

## 9. Heat Shock Proteins

Heat shock proteins have a protective role as counter-regulators of the inflammatory system, by reducing the production of proinflammatory cytokines. This may prolong survival time in major clinical syndromes [50].

### Acclimatization

**Figure 1:**
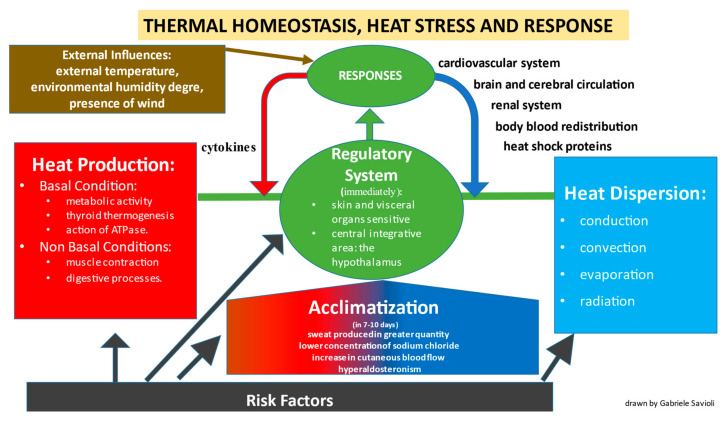
When the body is under heat stress, thermal homeostasis is maintained through the regulated balance (immediately for the regulatory system and after 7–10 days through the acclimatization process) among the factors that produce heat (red square) and heat dissipation (blue square). However, modifiable and non-modifiable risk factors can compromise both the regulation and acclimatization systems and the factors capable of producing or dispersing heat.

Acclimatization takes 7–10 days—its mechanisms remain largely unknown. It results in greater and earlier production of sweat. This can even quadruple and may start at a reduced core body temperature threshold. The electrolyte in the sweat may be altered by reducing its sodium concentration. There is also a steady increase in cutaneous blood flow. Hyperaldosteronism that occurs can cause a reduction in potassium levels (Figure 1).

## 10. Diagnosis and Management

Depending on how high the ambient temperature is and other environmental factors (e.g., humidity, ventilation), thermogenic factors, the functioning of heat dissipation mechanisms, and individual thermoregulation, which, in turn, are affected by various risk factors, heat illness manifests itself in a pathological clinical continuum from mild to major.

### 10.1. Mild Forms

Heat edema is a widespread symptom, characterized by gravitational soft-tissue edema, due to vasodilation and redistribution of body flows, typically involving the lower limbs. It is resolved by placing the legs in a supine position. Diuretics are not suitable for treatment of this type of edema [51].

Muscle cramps from heat: This is a common and benign form of heat disease. It manifests in short-term muscle cramps in the muscles subjected to stress. The cramps are often triggered by prolonged or intense physical activity, especially in athletes with incomplete physical training or in unacclimated workers. Excessive temperature is not necessary and often the subject’s temperature is also normal. Sweating is normal or excessive. The patient is lucid and may report thirst or fatigue. The onset of pain and cramps can be rapid or slow. Usually, anamnestic data are silent, and objectivity is non-specific. The most widely accepted etiological hypothesis is that of neuromuscular control theory [52,53]. The abdominal and lower limb muscles are the most involved [51]. Treatment includes appropriate isotonic fluid replacement, stretching, and massage (Table 2).

Heat rash Clogged pores trap sweat in the skin, causing erythematous papules and pustules. Clothed skin is most affected. Usually, by moving the patient to a cool environment, the rash ends. Sometimes it is necessary to remove excess clothing; the rash disappears after the skin has dried. These disorders are self-limiting and rarely require medical attention. After the symptoms have disappeared, the patient can return to the activity they were pursuing [54].

Tetany: Although often confused with muscle cramps, tetany can be distinguished from them by the appearance of perioral spasms, spasms of the feet and hands, and distal paresthesias.

### 10.2. Moderate Forms

Exercise-associated collapse (or heat syncope): This occurs very frequently and is characterized by high body temperatures. It can be due to a lack of water or salt which results in volume depletion. The first situation is typical for geriatric people, the second is characterized by hyponatremia and hypochloremia due to inadequate salt intake. Worsening factors are peripheral vasodilation and decreased vasomotor tone. It typically occurs immediately after strenuous exercise. It is characterized by symptoms that may precede syncope, such as agitation, confusion, thirst, asthenia, and headache [55,56]. Syncope is short-lived and resolves with supine posture, so treatment is largely supportive. Affected individuals must be placed in Trendelenburg position, the recovery of liquids or salts must be performed. Rest in a cool environment is recommended. Symptoms thus treated usually resolve within 15–20 min [57]. Exercise-associated collapse can be difficult to distinguish from cardiogenic syncope [8].

Heat exhaustion is the most common heat-related illness and is characterized by volume depletion under heat stress conditions. The heat stress effectively exceeds cardiac output. The symptomatology of this clinical picture is represented by weakness, headache, nausea, dizziness, myalgia, hypotension, tachycardia, hyperventilation, and muscle cramps. At blood test, there can be a hypernatremic condition, especially in intense physical stress, such as military personnel or athletes, or hyponatremia in subjects who presented intense sweating but were able to take only free water. If not recognized and treated it can evolve into heat stroke. Heatstroke differs because there are evident manifestations of central nervous system alteration, the temperature exceeds 41 °C and transaminases increase after 24 h. Since mental status remains intact in heat exhaustion, any alteration affecting the central nervous system must be considered heat stroke even if the temperature does not exceed 41 °C (Table 2).

Treatment includes placing the patient in a supine position in a cool environment. Cooling of the head with cold water, moisturizing of the skin, and placing of iced bags. Water and electrolytes need to be replaced either orally or intravenously. Potential complications include electrolyte disorders (e.g., hypernatremia, hypokalemia), rhabdomyolysis, mild hepatocellular injury, and acute renal failure. The presence of these findings is clinically related to burns and often requires hospitalization. For this reason, lab test include complete blood count, basal metabolism panel, liver function test, coagulation test, and measurements of creatine kinase and myoglobin level. Most patients who are stable with reassuring test results can be safely discharged after observation [58].

### 10.3. Severe Forms

Heat stroke is a medical emergency caused by failure of thermoregulation and acclimatization mechanisms. It is characterized by a rapid increase in internal body temperature that exceeds 40.5 °C, resulting in multi-organ dysfunction.

Basically, the The dysfunction of central nervous system, which leads to loss of consciousness, delirium, confusion, agitation, convulsions, and coma. The onset is usually sudden with symptoms of alteration of the central nervous system; there are rarely prodromal symptoms (Table 2).

The clinical picture is completed by warm skin with or without diaphoresis, hypotension, tachycardia, and tachypnea. Failure to sweat is a late manifestation. The organs most affected by high internal temperature are the brain and liver and the prognosis is related to the time spent in hyperthermia [31]. Although there is no cut-off temperature beyond which tissue damage occurs, many studies have shown that the severity of damage and cell death is related to the degree and duration of hyperthermia. Death can occur after the onset of heat stroke and is associated with heart failure, infact the first fundamental line of treatment is reduction in body temperature that help out to the support of the cardiovascular system. About one-third of patients who survive the initial damage experience multi-organ failure. In the event of pre-shock or shock, treatment should include the ABCDE scheme with maintenance of the airways, respiration, and circulation, followed by rapid cooling. If cooling is completed within 30 min of collapse, the mortality rate approaches zero [59,60]. Patients who present to the emergency department with an internal body temperature of 41 °C or higher and prolonged hyperthermia have mortality rates of up to 80%.

We distinguish two types of heatstroke: classic heatstroke and stress heatstroke. The classic type affects elderly people, children, or people of low economic status who live in unsuitable environments. It is diagnosed where the body temperature of the subjects is higher than 40.5 °C. In these cases, the beginning and evolution of the clinical picture can be deceptive. Frequently, there is dehydration and coma. The second category is hot exercise heatstroke. In this case, the affected subjects are younger, often athletes or military personnel, who have been engaged in intense physical activity. Coagulation disorders and rhabdomyolysis and latex acidosis are more frequent. Mortality can reach 50% of cases. In exercise heatstroke, the temperature is usually below 40.5 °C.

In both cases, it is possible to detect leukocytosis, thrombocytopenia, impaired coagulation, liver damage, renal damage, and rhabdomyolysis in the blood chemistry.

Where there is a suspicion of heat stroke, it is necessary to reduce the temperature as quickly as possible. In fact, as we have already discussed, mortality and morbidity are dependent on the duration and severity of hyperthermia. Clothes should be promptly removed, and the patient immersed in cold water (46° to 57° F [8° to 14 °C]) or ice water (35.6° to 41°F [2° to 5 °C]), which can cool down by 0.26 °C per minute and up to 0.35 °C per minute, respectively [59,61]. Continuous monitoring of core rectal temperature is recommended during rapid cooling. There is no temperature cut-off value at which to stop cooling [51,62,63,64]. Where the patient cannot be immersed in water, wet towels, ventilators, or water nebulizers can be used, with less effectiveness, especially in pre-hospital environments where it is necessary to use any available means, in the absence of the most appropriate ones. If, on the other hand, the most effective techniques are readily available in the pre-hospital environment, it is necessary to follow the rule “cool first and transfer later”, and continue cooling during transport with available means [14,55,59,60,64]. For inpatient treatment of heat stroke, emergency room procedures will apply.

**Table 2:** Clinical patterns of heat disease.

Mild Form	Moderate Form	Severe Form
Heat edema	Exercise-associated collapse (or heat syncope)	Classic heatstroke
Muscle cramps from heat	Hypernatremic heat exhaustion	Exertional heatstroke
Heat rash	Hyponatremic heat exhaustion	
Tetany		

## 11. Prevention

In most cases, the onset of heat-related disease can be avoided or its symptoms reduced to a mild level [55,65]. It is necessary to maintain adequate hydration, to wear loose and light-colored clothing and to avoid the hottest hours, and to perform activities only in the most appropriate hours (with the lowest temperatures) of the day. In the case of travel or movement to very hot places, it is necessary to allow complete acclimatization. When it is not possible to avoid the hottest hours or activity at high temperatures, frequent breaks for hydration, scheduled rest and recovery cycles, as well as careful monitoring, are recommended. Particular attention should also be paid to fragile categories of person, for whom these recommendations are mandatory. Children and the elderly who may not have symptoms of thirst or be unable to hydrate themselves should be hydrated.

## 12. Point of View of the Emergency Department

### 12.1. General Aspects

In the hottest periods, patients with heat disease may be admitted to emergency departments. Since it is often difficult to perform anamnestic triage [66], especially in crowded conditions, where the outcomes are worse per se [67,68,69], it is necessary to pay close attention to risk factors, especially those related to pathologies. Risk factors for developing heat diseases help to identify fragile populations, such as the elderly, who already suffer worse outcomes in emergency departments [70,71,72,73,74]. It is necessary to pay particular attention to these patients, often using multiple drugs, which is often underestimated in triage. In the most fragile categories, and in the most compromised patients, it is useful to perform blood chemistry tests, including blood counts, and analyses of biochemistry, liver enzymes, electrolytes, renal function, and CPK dosage. An ultrasound evaluation will allow the estimation of ventricular filling pressures and the patient’s volume status [75,76,77]. Patients with heat exhaustion or presenting major clinical pictures should be treated and stabilized in observation units, which have been found to be effective for large categories of patients [78,79]. Producing plans for any hyper-influxes of patients in hot seasons can be important, especially if they include major sporting or entertainment events, as has been demonstrated in other fields [80].

### 12.2. Inpatient Treatment of Heat Stroke

In the emergency room, such patients should be monitored and treated immediately.

Acute management of hyperpyrexic patients includes evaluation and stabilization of airways, breathing and circulation, placement of a rectal thermometer for accurate temperature monitoring and, the mainstay of hyperthermia management, rapid cooling. As the initial survey is conducted and cooling therapy is initiated, patients should be attached to continuous cardiac monitoring, a foley catheter placed for accurate monitoring of urine output, pertinent laboratory work initiated, and a 12-lead electrocardiogram and intravenous access should be obtained. There are several methods of rapid cooling, but noninvasive methods, such as evaporative cooling and ice-water immersion are the most evidence-based and widely used. Ice-water immersion is considered by some to be the gold standard for cooling therapy and can cool at a rate of 0.15 °C/min, but this can be difficult to accomplish in an emergency department and may limit patient-monitoring capabilities and concurrent resuscitation efforts [81]. Evaporative cooling, which uses fans to blow warmed air (45 °C) over the patient as they are sprayed with cooled, atomized water (15 °C) has a cooling rate of 0.08 °C/min [82]. This produces a slightly slower rate of cooling but with fewer impracticalities and a decreased shivering response. Owing to their lesser cooling efficacies, other therapies can be used as adjuncts but should not be used alone. These include ice-packing (either whole-body or strategically at the neck, axillae, and groin), use of cooling vests or blankets, cooled humidified oxygen or cooled intravenous fluids, or more invasive methods, such as cold gastric or rectal lavage. Peritoneal lavage has a marked benefit with a cooling rate of up to 0.5 °C/min but is invasive and should not be the first-line of therapy if non-invasive methods can be effective. Cooling should be discontinued after temperature measured by a rectal probe reaches 39 °C to prevent overshooting normothermia [83].

If cooling is the key therapy for heat stroke, it should be emphasized how important it is to also support the circulation; the absence of adequate hemodynamic support is linked to a high rate of death and disabling neurological damage. Echocardiographic monitoring can help identify patients with a hyperdynamic response from those with a hypodynamic response. The former has a high cardiac index, low resistance, and high central venous pressure secondary to right heart failure. It is necessary to be cautious in the volemic expansion of these patients because the cooling itself causes vasoconstriction and an increase in systemic pressure. Patients with a hypodynamic response, on the other hand, have a low cardiac index, high central venous pressure, and high systemic and pulmonary resistance. In these cases, a low dose of dobutamine may be useful.

The infusion of cold saline solution can enable reduction in the number of days of hospital stay. Adequate hydration with cold fluids has also been shown to limit kidney damage and normalize liver damage more quickly [84]. Antipyretic drugs are not initiated in heatstroke [85]. Shivering or muscle cramping, when they occur, can be managed with intravenous diazepam or chlorpromazine [86]. Once the critical neurological phase has been overcome and the temperature has been restored, the patients must be monitored and blood tests repeated initially and at 24 h to assess the risk of developing rhabdomyolysis, acute respiratory distress syndrome, compartment syndrome, liver dysfunction, acute renal failure, electrolyte abnormalities or disseminated intravascular coagulopathy.

One of the most important mediators of heatshock toxicity is the systemic inflammatory response [87]. A therapy that removes inflammatory mediators and other heatshock-invoked toxic substances should improve recovery from this condition. Continuous renal replacement therapy (CRRT) can reduce the inflammatory response, clear toxic metabolites, rectify water levels, electrolyte, and acid/base imbalance, and maintain homeostasis [88]. Although studied extensively for the treatment of sepsis [89,90], its potential usefulness in HS therapy has not been thoroughly investigated.

Observation and stabilization for 24 h in the observation unit at a medium-high intensity of care is recommended if the emergency department has these facilities, and therefore, hospitalization is required for treatment and observation.

## 13. Differential Diagnosis

Physicians should consider other potential conditions in differential diagnosis for patients with hyperthermia and altered mental status [83]. There is no single laboratory test that can confirm or exclude the diagnosis of heatstroke. Laboratory investigations include complete blood count, kidney function, liver function, and electrolytes [91,92,93,94,95]. Laboratory features that support the diagnosis of heatstroke include leucocytosis, which can reach 30–40 × 10^3^/mm^3^, increased urea and creatinine, increased serum transaminases, increased creatinine kinase, hyponatremia, metabolic acidosis, and respiratory alkalosis; myoglobinuria, hemogranular cast, proteinuria. Polycythaemia, hypercalcemia, and hyperalbuminemia occur due to dehydration. The condition of hypokalaemia and hypophosphatemia result from loss of perspiration, the effects of catecholamines, and hyperventilation [96]. Hyperglycaemia is often associated with non-exertional heatstroke (NEHS), whereas hypoglycemia, although rarely occurring, tends to occur in exertional heatstroke (EHS) [97]. The clinical features of heatstroke can mimic other medical conditions, such as meningitis, encephalitis, malaria, malignant neuroleptic syndrome, hyponatremia, septic shock, thyroid crisis, acute myocardial infarction, malignant hyperthermia, and drug use or reactions [98,99,100,101]. Hyponatremia can be distinguished from heatstroke by a history of excessive fluid consumption, normal pulse, normal body temperature, polyuria, and normal or hypertension [96]. Malignant hyperthermia results from increased heat production by muscle hypermetabolism after exposure to anesthetic drugs. Malignant neuroleptic syndrome is an idiosyncratic reaction to dopamine antagonists resulting from a combination of muscle activity and inhibition of heat loss [102]. Toxicological screening is useful to exclude drugs that can cause hyperthermia: ethanol, amphetamines, cocaine, salicylates, hallucinogens and lithium.

## 14. Post-Mortem Investigations in Cases of Death by Hyperthermia

### 14.1. Traditional Post-Mortem Examinations

Hyperthermia represents a challenge for the forensic pathologist. The autopsy usually reveals only unspecific findings and only a few studies have been conducted on this topic. The suspicion of heat-stroke-related death could be raised by a surprisingly high rectal temperature, even if this is true only when the body examination is performed soon after death. Bunai et al. described the case of a man who died of hyperthermia whose rectal temperature a few hours after death was 40 °C [103]. It is essential to properly evaluate the investigation scene (ambient temperature in particular), circumstantial data, and subject characteristics to determine if there are conditions for a lethal increase in body temperature. Hypostasis and rigor mortis are typically enhanced by higher temperature, so they could be more precocious and severer in this kind of death, but these data are useful only if the time of death is known. Vice versa, along with the higher rectal temperature, they represent a confounding factor in the determination of the time since death. If the survival time after onset of heatstroke is short, polyvisceral congestion may be the only finding. Petechial hemorrhages of the skin and inner organ surfaces, as well as hemorrhages in the spleen, lymph nodes, and thymus, have been described [103,104]. Unspecific signs of tissue damage are usually present, such as brain edema and diffuse neuronal injury, centrilobular necrosis, and acute pancreatitis, especially when the victim survived longer (>12–24 h) [105,106,107]. If rhabdomyolysis occurred, acute tubular necrosis may be found. The pathologist should also look for thrombi and microthrombi to rule out disseminated intravascular coagulation [108].

### 14.2. Immunohistochemistry

Immunohistochemistry could be a supplemental investigation in such cases. Fineschi et al. described the case of an eight-day-old infant who died of heat stroke [109]. They demonstrated that heat shock proteins (HSP) 70, 27, and 90 immunopositivity in the trachea and skin epithelium was higher than in normal tissue. Some authors have observed that the expression of adrenocorticotropic hormone (ACTH) in the pituitary gland, ubiquitin and myoglobin in the renal tubular epithelium, and in the hypothalamus, dopamine were lowered, while the expression of dopamine and noradrenaline in the adrenal medulla, ubiquitin in the periaqueductal gray matter of the midbrain, and chromogranin A in the adrenal medulla, were increased in cases of hyperthermia [110,111,112,113].

### 14.3. Biochemical Analyses

Other useful investigations in such kinds of death are biochemical analyses. As previously mentioned, a consequence of severe hyperthermia is dehydration. Therefore, electrolyte abnormalities are often present. Post-mortem sodium and chloride levels in the vitreous humor may be higher than usual, indicating ante-mortem hemoconcentration due to loss of body water [108]. Accordingly, other studies have shown that creatinine levels are elevated in the serum and pericardial fluid [104,114,115]. On the other hand, it appears that C reactive protein serum level is decreased in such deaths [116,117]. Cardiac markers, such as cardiac troponin T and I, creatinine kinase MB, and atrial and brain natriuretic peptides (ANP and BNP) levels could also be elevated in serum and pericardial fluid [118,119]. Kortelainen et al. demonstrated that urinary noradrenaline concentration is increased in hyperthermia-related deaths [120]. A possible explanation is that heat stress stimulates the sympathetic system. This finding was indirectly confirmed by further research, which demonstrated that postmortem catecholamine serum levels were also elevated [112]. However, these biochemical investigations are not specific to heat stress and hyperthermia-related death, since variation in such parameters can also be found in cases of death for other causes. Therefore, a postmortem diagnosis of fatal hyperthermia should always be based on a series of findings that need to be contextualized for the circumstances of death.

## Data Availability

Not applicable.
